# Heterogeneity in Arterial Remodeling among Sublines of Spontaneously Hypertensive Rats

**DOI:** 10.1371/journal.pone.0107998

**Published:** 2014-09-24

**Authors:** Erik N. T. P. Bakker, Gergely Groma, Léon J. A. Spijkers, Judith de Vos, Angela van Weert, Henk van Veen, Vincent Everts, Silvia M. Arribas, Ed VanBavel

**Affiliations:** 1 Department of Biomedical Engineering and Physics, Academic Medical Center, Amsterdam, the Netherlands; 2 Department of Vascular Medicine and Nephrology, Academic Medical Center, Amsterdam, the Netherlands; 3 Van Leeuwenhoek Center for Advanced Microscopy, Department of Cell Biology, Academic Medical Center, Amsterdam, the Netherlands; 4 Departamento de Fisiología, Facultad de Medicina, Universidad Autónoma de Madrid, Madrid, Spain; Universidade Federal do Rio de Janeiro, Brazil

## Abstract

**Objectives:**

Spontaneously hypertensive rats (SHR) have been used frequently as a model for human essential hypertension. However, both the SHR and its normotensive control, the Wistar Kyoto rat (WKY), consist of genetically different sublines. We tested the hypothesis that the pathophysiology of vascular remodeling in hypertension differs among rat sublines.

**Methods and Results:**

We studied mesenteric resistance arteries of WKY and SHR from three different sources, at 6 weeks and 5 months of age. Sublines of WKY and SHR showed differences in blood pressure, body weight, vascular remodeling, endothelial function, and vessel ultrastructure. Common features in small mesenteric arteries from SHR were an increase in wall thickness, wall-to-lumen ratio, and internal elastic lamina thickness.

**Conclusions:**

Endothelial dysfunction, vascular stiffening, and inward remodeling of small mesenteric arteries are not common features of hypertension, but are subline-dependent. Differences in genetic background associate with different types of vascular remodeling in hypertensive rats.

## Introduction

Since its development in 1963 by Okamoto [1] the spontaneously hypertensive rat (SHR) has been used in more than 17,000 publications as a model of human essential hypertension. Resistance arteries from SHR and its normotensive control strain, the Wistar Kyoto rat (WKY), have been compared extensively. This has resulted in the observation that vessels from SHR are remodeled, as evidenced by an increase in the wall-to-lumen ratio [2], [3]. Herein both a thickening of the wall and a reduction in lumen diameter contribute potentially. Such remodeling is also observed in human hypertension, strengthening the SHR as a useful model for human hypertension [2]. While vascular remodeling can be seen as an adaptation to the increased pressure, clinically an increased wall-to-lumen ratio predicts cardiovascular events independent of blood pressure [4], [5].

An increase in wall-to-lumen ratio appears a consistent finding in SHR. However, true inward remodeling, i.e. a reduction in lumen diameter, is not. This is important since even minor reductions in lumen diameter at the level of small arteries and arterioles greatly reduce maximal perfusion capacity and contribute to the maintenance of high blood pressure. Thus, while some papers show a clear reduction in lumen diameter in vessels from SHR as compared to WKY,[6]–[8] others did not find this in arteries from the same vascular bed [9], [10]. Similarly, the question whether hypertension is associated with vascular stiffening has yielded completely opposite results for SHR. Thus, some studies show a clear stiffening, as indicated by analysis of stress-strain relationships of vessels from SHR and WKY [8], [11], whereas others found a profound reduction in stiffness in SHR as compared to WKY [7]. This inconsistency is not limited to the microcirculation, but also seen in large arteries [12]–[15]. Finally, also the association of hypertension with endothelial dysfunction remains ambiguous. Endothelium-dependent relaxation in SHR and WKY has been tested in many studies, with an inconsistent outcome [16].

While data in literature may appear contradictory because of differences in methodology and other factors, the fact that WKY rats from different sources differ markedly was already shown by early work of Kurtz [17]. This is probably because the WKY was distributed by the NIH before it was fully inbred. Progress in the field of genetics has revealed that sublines of SHR also exist, and that they display different sensitivity to hypertension-related end organ disease [18]–[20]. In WKY and SHR sublines, up to 2.5% difference was found among 10,000 single nucleotide polymorphisms that were tested [20]. We therefore hypothesized that the pathophysiology of hypertensive remodeling differs among sublines of rats. To address this hypothesis, we studied WKY and SHR rats, including the stroke-prone SHR, from three different sources. With this approach we aimed not only to provide an explanation for discrepant data in literature, but also to gain further insight in the pathogenesis of hypertensive vascular remodeling.

## Materials and Methods

### Animals

Male SHR and WKY rats at 6 weeks and 5 months of age (n≥5 per sub-line and time point) were obtained from three different locations. The SHR/NCrl, WKY/NCrl, and stroke-prone SHR animals (SHRSP/A3NCrl) rats were obtained from Charles River, the Netherlands. The SHR/NHsd and WKY/NHsd rats were derived from Harlan UK, (Bicester, U.K.) and bred at the local animal facility of the Universidad Autónoma de Madrid, Spain. WKY/NTac rats were obtained from Taconic Farms (Germantown, NY, USA). All experiments were approved by the Committee for Animal Experiments of the Academic Medical Centre, Amsterdam.

### Blood pressure

After a one-week training period, in which the rats were acclimatized to handling and the restrainer, systolic blood pressure (SBP) was measured using the tail-cuff method. For each rat, at least three successive measurements were recorded and averaged.

### Tissue collection

Animals were anesthetized with isoflurane (5%), weighed, heparinized, and blood was taken by a heart puncture. Animals were then sacrificed by decapitation. The intestines were removed en block and placed in chilled MOPS buffer. Second- and third-order branches of the mesenteric artery were dissected using a stereomicroscope. From each animal, two vessels were used for analysis in a pressure myograph setup, of which some were further processed for electron microscopy. One segment was used for wire myography. A segment of the thoracic aorta was also dissected, in order to determine the force-distension relationship, using a wire myograph setup. In addition, sections of the aorta were cut and stained to determine wall thickness and wall cross sectional area (CSA). With these data, stress-strain relationships were calculated.

### Pressure myography

To determine the passive properties of the mesenteric resistance arteries, segments from 6 week and 5 months old rats were cannulated and mounted in a pressure myograph setup. From each animal two vessels were tested and data were averaged. The vessels were unbuckled by the adjustment of the cannulas at 100 mmHg intraluminal pressure and the inner and outer diameters were recorded. Diameters were determined at 5, 25, 50, 75, 100, 125, 150 and 175 mmHg of intraluminal pressure in calcium-free MOPS buffer, in the presence of 0.1 mmol/L papaverine to abolish vasomotor tone, at 37°C. Wall-to-lumen ratios were calculated at 100 mmHg intraluminal pressure. Circumferential strain (ε) was calculated as the diameter ((Dx-D5)/D5) were D5 is the diameter at 5 mmHg and Dx is the diameter at a given pressure. Circumferential stress (σ) was calculated as (P·r)/h, were P stands for pressure, r stands for radius and h for wall thickness. Stress-strain relationships were fitted using the equation: σ = E_el_ ⋅ ε+E_col_ ⋅ max (ε–ε_ho_, 0). This yielded three parameters, representing the slope of the first part of the relationship (E elastin), the slope of the second part of the relationship (E collagen) and the intersection with the x-axis, i.e. the level of strain where collagen is proposed to hook-on (ε hook-on) [21].

### Endothelium-dependent relaxation

Endothelial function was tested at five months of age. Mesenteric arteries were mounted in a wire myograph setup, filled with physiological salt solution (composition in mM: 119 NaCl, 25 NaHCO_3_, 4.7 KCl, 1.18 KH_2_PO_4_, 1.17 MgSO_4_, 1.6 CaCl_2_, 0.027 EDTA, 5.5 glucose; pH 7.35) at 37°C and gassed with 95% air and 5% CO_2_. Vessels were first normalized, and then exposed to 125 mmol/L potassium solution to test for viability. Subsequently, arteries were pre-contracted with 3⋅10^−7^ mol/L thromboxane analogue U46619, and exposed to increasing doses of methacholine to test endothelium-dependent relaxation.

### Electron microscopy

For ultrastructural analysis, mesenteric resistance arteries of 5-months old rats were used. From each sub-line, arteries from three animals were cannulated in calcium-free MOPS buffer, fully dilated with 0.1 mmol/L papaverine, and pressurized to 100 mmHg. Vessels were then fixed for 1 hour in 1% glutaraldehyde and 4% paraformaldehyde in 0.1 mol/L Na-cacodylate buffer (pH 7.4). After the fixation under pressure the vessels were stored in the same fixative without papaverine at 4°Celsius. The vessels were then washed in distilled water, postfixed for 60 min. in 1% OsO4 in water and washed again in distilled water. For contrast enhancement the vessels were block stained overnight in 1.5% aqueous uranyl acetate, dehydrated through a series of ethanol concentrations and embedded in resin LX-112 (Ladd), The resin blocks were polymerized for 48 hours at a temperature of 60°C. Ultrathin sections of 90 nm were cut on a Reichert EM UC6 with a diamond knife, collected on formvar coated grids and stained with uranyl acetate and lead citrate. Sections were examined with a FEI Technai-12 G2 Spirit Biotwin electron microscope. Images were taken with a Veleta camera (Olympus, SIS). Images of the cross sections were taken at a magnification of ×300 and at ×4,500 magnification for longitudinal sections of RMAs. For collagen diameter measurements images were taken at a magnification of ×60,000. All quantifications on electron micrographs including quantification of smooth muscle cell layers, internal elastic lamina thickness and collagen diameter measurements were done by using Item software (Olympus Soft Imaging Solutions).

### Immunohistochemistry

Frozen, embedded mesenteric arteries obtained from rats at 5 months of age, were cut into 5 µm sections and fixed for 20 min in 100% methanol. Following a blocking step in 3% bovine serum albumin +0.005% triton X, cross sections were incubated overnight with the primary antibody at 4°C, washed, and incubated with the relevant secondary antibody for one hour at room temperature. Samples without primary antibodies were used as negative control. The primary antibodies used were: rabbit anti-collagen I (Millipore – AB755P; 1∶400), mouse anti-collagen III (Abcam – ab23445; 1∶400), rabbit anti-collagen V (Millipore – AB2038; 1∶400), rabbit anti-collagen VI (Abcam – ab6588; 1∶400), and rabbit anti-decorin (Bioss – bs1695R; 1∶200). As secondary antibody either Cy-3 labelled goat anti-rabbit antibody (Brunschwig 111-165-144) or goat anti- mouse (Brunschwig –115-165-166) was used in a 1∶300 dilution with 1% bovine serum albumin. To visualise nuclei, bisbenzimide (3.5 mg/ml) at a 1∶100 dilution for 3 min, was used. Sections were covered with DAKO fluorescent mounting medium (DAKO – S3023) and coverslipped.

### Statistics

Data are presented as mean ± SEM. Statistical analysis was performed using SPSS software (version 20). Differences between WKY and SHR were tested using one-way ANOVA or repeated measurements ANOVA where appropriate. Post-hoc analysis between sublines was done using Tukey’s test.

## Results

### Heterogeneity in body weight and blood pressure among WKY and SHR sublines

When averaged over all substrains, the body weight of WKY and SHR was not different at the age of 6 weeks. At 5 months of age, WKY were significantly heavier than SHR (Table 1). Among WKY, the WKY/NTac stood out as its body weight was about twice that of the other two sublines at 6 weeks of age. At 5 months of age, the WKY/NTac was still much heavier than the other sublines. Among SHR, differences in weight were more subtle, but statistically significant in several cases (see Table 1).

**Table 1:** Body weight, vessel diameter and remodeling indices.

	WKY/NCrl		WKY/NHsd		WKY/NTac		All WKY	
	6 wk	5 mo	6 wk	5 mo	6 wk	5 mo	6 wk	5 mo
**Body weight (g)**	121±6	435±5	113±6	378±5*	244±9†‡	580±9†‡	152±14	452±14
**Lumen diameter (µm)**	285±9	363±18	319±9	383±5	391±24†	417±11	332±14	386±9
**Wall thickness (µm)**	20±1	18±1	20±3	24±1*	20±1	20±1	20±1	20±1
**Wall CSA (x1000 µm^2^)**	19±1	22±1	20±2	31±1*	26±2†	27±1†‡	22±1	26±1
**Wall/lumen ratio (%)**	6.9±0.4	4.9±0.4	6.2±0.8	6.3±0.3*	4.9±0.3†	4.7±0.2‡	6.0±0.4	5.3±0.2
	**SHR/NCrl**		**SHR/NHsd**		**SHR/SP**		**All SHR**	
	**6 wk**	**5 mo**	**6 wk**	**5 mo**	**6 wk**	**5 mo**	**6 wk**	**5 mo**
**Body weight (g)**	148±4	365±5	112±3*	394±14*	130±4§¥	359±8¥	131±4	369±5#
**Lumen diameter (µm)**	319±13	367±8	300±9	354±22	307±8	369±7	308±6	364±7
**Wall thickness (µm)**	21±1	28±2	25±1	32±1	21±1	28±2	22±1#	29±1#
**Wall CSA (x1000 µm^2^)**	23±1	35±2	26±1*	37±3	22±2	35±2	24±1	36±1#
**Wall/lumen ratio (%)**	6.6±0.5	7.6±0.6	8.5±0.3*	9.1±0.7	6.8±0.4¥	7.7±0.5	7.3±0.3#	8.1±0.4#

Body weight and vascular parameters were measured at 6 weeks and 5 months of age. Note the weight of WKY/NTac rats is twice that of the other WKY sublines at 6 weeks of age. Other parameters were measured on cannulated mesenteric arteries at 100 mmHg under fully dilated conditions. Each group consists of n≥5 animals. From each animal 2 vessels were measured and averaged. Data are mean ± SEM. Symbols indicate P-value <0.05, corrected for multiple testing using Tukey’s post hoc test.

*NCrl vs. NHsd, †NCrl vs. NTac, ‡NHsd vs. NTac of the same age.

*NCrl vs. NHsd, §NCrl vs. SP, ¥NHsd vs. SP, #WKY vs. SHR of the same age.

**Figure 1:**
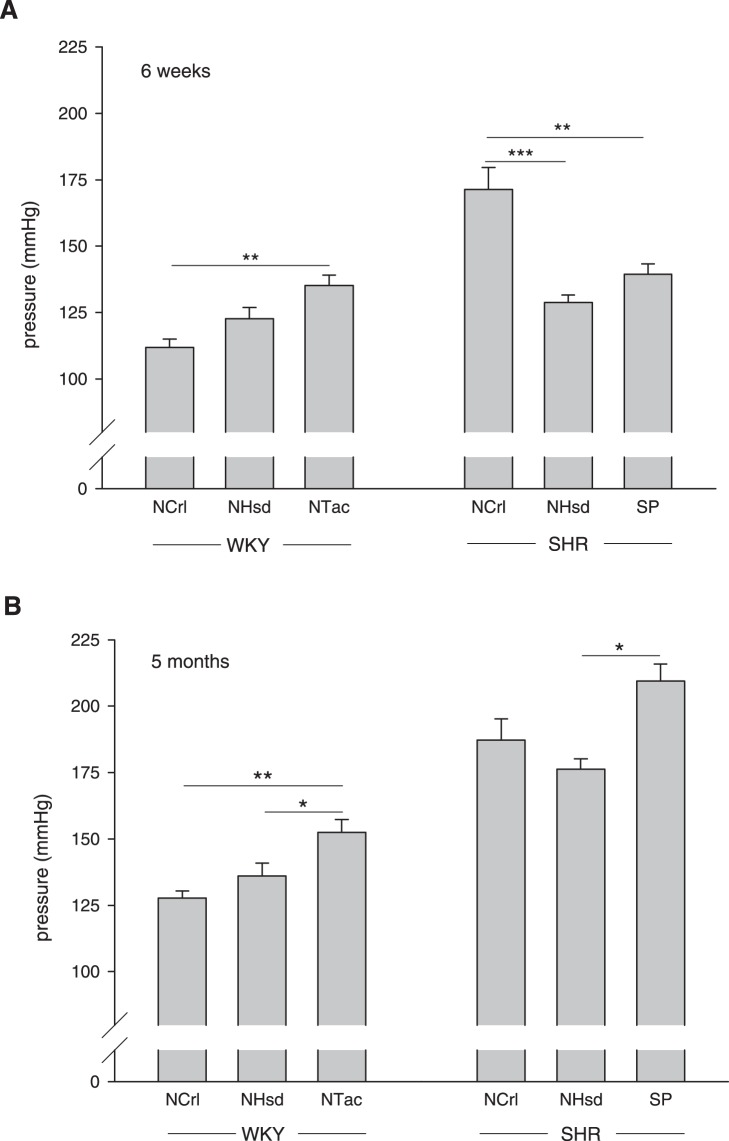
Blood pressure. Systolic blood pressures were measured using the tail cuff method at 6 weeks (panel A) and 5 months of age (panel B). Large differences between sublines were recorded in the level and development of blood pressure over time. Data are mean ± SEM with WKY/NCrl 6 w: n = 5 and 5 mo: n = 6; WKY/NHsd 6 w: n = 6 and 5 mo: n = 4; WKY/NTac 6 w: n = 5 and 5 mo: n = 5; SHR/NCrl 6 w: n = 5 and 5 mo: n = 6; SHR/NHsd 6 w: n = 6 and 5 mo: n = 4; SHR/SP 6w: n = 6 and 5 mo: n = 6. *Indicates P<0.05, ** P<0.01 and *** P<0.001.

Systolic blood pressure was measured using the tail cuff method. At 6 weeks of age, the SHR (all sublines included) already showed a significant increase in blood pressure as compared to WKY: 123±3 mmHg for WKY and 145±5 mmHg for SHR (P = 0.001). At 5 months of age, a more pronounced elevation in blood pressure was measured: 138±4 mmHg for WKY and 193±5 mmHg for SHR (P<0.001). Among WKY sublines, the WKY/NTac had a higher blood pressure than the WKY/NCrl at both ages, and a higher blood pressure than the WKY/NHsd at 5 months of age. Among SHR, the SHR/NCrl demonstrated a markedly higher blood pressure at 6 weeks of age (Figure 1A), whereas at 5 months of age the SHR/SP was the most hypertensive subline (Figure 1B).

### Vascular remodeling

To study vascular remodeling, small mesenteric arteries were cannulated and pressurized in a myograph setup. Using an automated wall tracking algorithm, the lumen diameter and wall thickness were measured over a range of pressures (5–175 mmHg). The wall cross sectional area (CSA) and wall-to-lumen ratio were subsequently calculated. Data for vessel dimensions are given in Table 1. Lumen diameter at 100 mmHg was not significantly different between WKY and SHR at both ages, although a tendency for smaller diameters was present in SHR at 5 months of age (P = 0.07). Among WKY, mesenteric arteries from the WKY/NTac stood out with relatively large lumen diameters as compared to the other WKY sublines, particularly at 6 weeks of age.

Wall thickness of mesenteric arteries was increased in SHR as compared to WKY, both at 6 weeks (P<0.05) and 5 months of age (P<0.001). The wall CSA was significantly increased in SHR as compared to WKY at 5 months of age only (P<0.001), whereas the wall-to-lumen ratio was increased in SHR as compared to WKY at both ages (P<0.001). Among both WKY and SHR sublines, significant differences were found in wall thickness, wall CSA, and wall-to-lumen ratio of small mesenteric arteries (Table 1).

**Figure 2:**
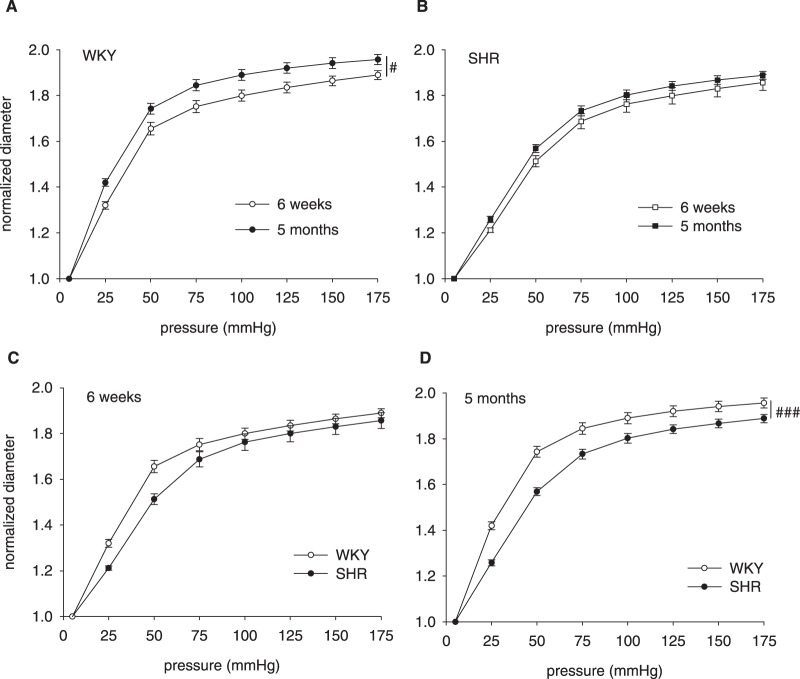
Vascular distensibility. Diameters were measured on cannulated mesenteric arteries, under fully dilated conditions. Diameters are normalized to the lowest pressure level to eliminate differences in vessel size. Panel A: WKY vessels show an increase in distensibility with maturation (5 months vs. 6 weeks), whereas SHR vessels do not (panel B). Panel C shows that at 6 weeks of age, distensibility of SHR vessels tends to be lower than vessels from WKY, but does not reach statistical significance. Panel D shows that at 5 months of age, SHR vessels are less distensible than WKY vessels. Data from individual sublines are shown in Figure S1. Data are mean ± SEM, with WKY 6w: n = 15 and 5 mo: n = 17; SHR 6w: n = 16 and 5 mo: n = 16. # Indicates P<0.05, and ### P<0.001.

Distensibility of mesenteric arteries was determined by normalizing diameter measurements to the diameter at 5 mmHg (Figure 2). Overall, WKY vessels became more distensible with maturation (P<0.01). Thus, repeated measurement analysis showed a significant difference between 6 weeks (Figure 2A) and 5 months (2B) of age in WKY. This did not occur in SHR vessels.

At 6 weeks of age (2C), distensibility of WKY and SHR vessels was not significantly different, although SHR vessels tended to be less distensible (NS; P = 0.08). At 5 months of age (2D), the SHR vessels were clearly less distensible than WKY (P<0.001). Among WKY sublines, significant differences were found between WKY/NCrl and WKY/NHsd at 6 weeks of age (Figure S1). Among SHR sublines, data were not significantly different at this time point. At 5 months of age, WKY sublines were not different. However, among SHR sublines the SHR/NCrl mesenteric arteries were more distensible than those of both other sublines (Figure S1).

### Vascular stiffness

**Figure 3:**
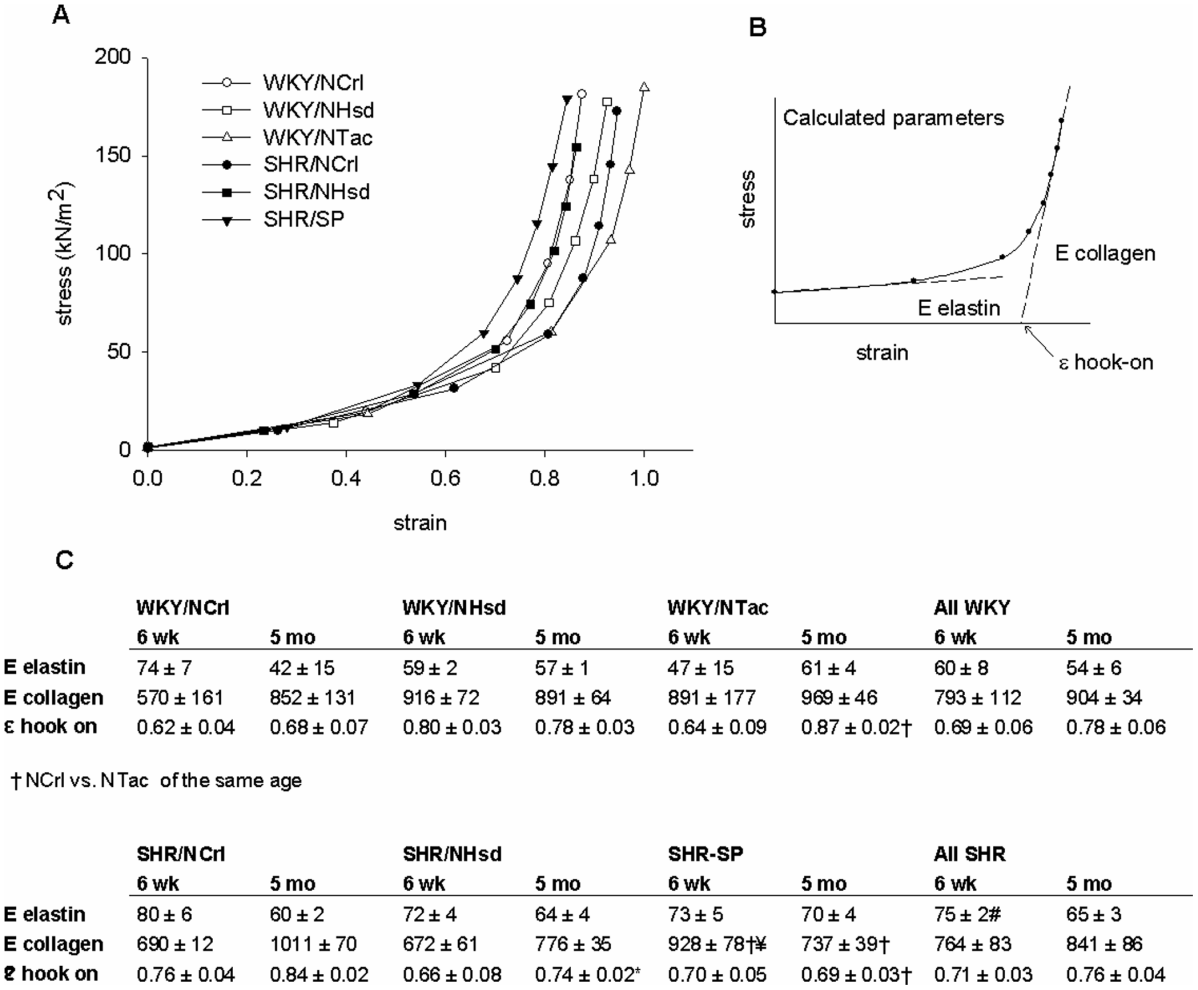
Vascular stiffness. To assess vascular stiffness, stress-strain relationships were calculated from diameter, wall thickness and pressure. Panel A shows that SHR vessels are not necessarily stiffer than WKY vessels. As illustrated in panel B, data were fitted to a three parameter equation to estimate the elasticity of elastin (E elastin, in kN/m2), the elasticity of collagen (E collagen, in kN/m2) and the level of strain were collagen is proposed to hook-on (ε hook-on). This revealed that the vessels from the SHR/SP are the most left in the stress-strain relationship because of an early hook-on of collagen (panel C). Each group consists of n≥5 animals, where 2 vessels from each animal were measured and averaged.

To determine the material properties of the arteries, stress-strain relationships were calculated on the basis of the applied pressure, the recorded diameter, and wall thickness. This relationship reflects the intrinsic mechanical properties of the vessel wall, taking differences in size or wall thickness out of the equation. In general, there was an overlap of WKY with SHR sublines, indicating that there is no consistent relationship between blood pressure and arterial wall stiffness in mesenteric arteries (Figure 3A). The stress-strain relationship is characterized by a shallow part at low strain that is attributed to elastin (E elastin), followed by a steep rise in stress (E collagen) where collagen is believed to ‘hook-on’ (ε hook-on; Figure 3B). The slopes of both parts of the stress-strain relationships and the level of strain that is associated with the hook-on of collagen were determined for each group (Figure 3C). This showed that E elastin is higher in SHR at 6 weeks of age, suggesting increased stiffness of the elastic component of the vessel wall. The values for E collagen and ε hook-on were not significantly different between WKY and SHR, but differences were found among sublines. Comparison of the WKY/NTac and the SHR/SP, being the groups with the lowest and highest stiffness respectively, shows that these features associate with a paradoxically low E collagen, but early collagen hook-on in the vessels of the SHR/SP. In other terms, this suggests that mesenteric arteries from the SHR/SP are not stiff and poorly distensible because of an intrinsically stiff collagen fraction, but rather because of a relatively early recruitment of collagens with distension.

### Aortae

The data of the aortae resembled those of the mesenteric arteries to a large extent. Thus, wall stiffness, reflected by stress-strain relationships, was not different between WKY and SHR aortae at the age of 5 months (Figure S2). Similarly, the size of the aortae was not different between WKY and SHR. Only the wall-to-lumen ratio was significantly higher in SHR as compared to WKY. Among the WKY sublines, the WKY/Tac stood out with a larger aortic diameter and wall CSA, which likely relates to the larger size of the animals. Among the SHR sublines, the SHR/SP showed a larger wall CSA and wall-to-lumen ratio as compared to the other SHR sublines (Figure S2).

### Endothelial function

**Figure 4:**
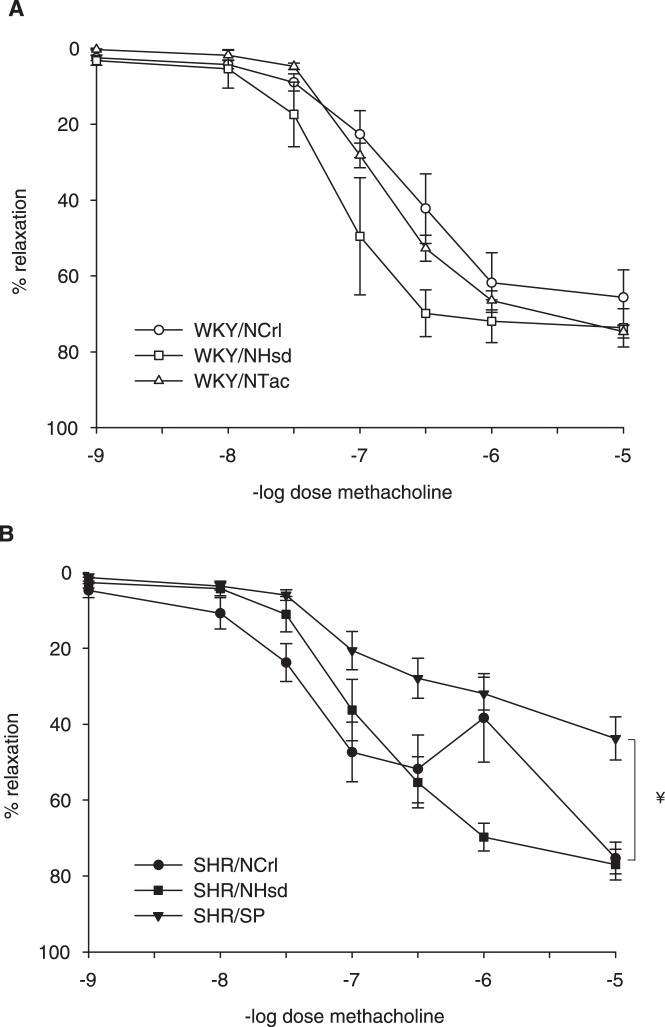
Endothelial function. To test endothelium-dependent relaxation, vessels of WKY sublines (panel A) and SHR sublines (panel B), were mounted in a wire myograph and contracted with 3⋅10^−7^ mol/L thromboxane analogue U46619. Then, increasing doses of methacholine were added to the vessel bath. Data are mean ± SEM with n≥5 animals. † Indicates P<0.05 for SHR/NCrl vs. SHR/SP. ¥ Indicates P<0.05 for SHR/NHsd vs. SHR/SP.

To determine endothelial function, mesenteric arteries from 5 months old rats were mounted in a wire myograph setup, pre-contracted with U46619 and exposed to increasing doses of methacholine. All WKY sublines showed a dose-dependent relaxation, with no significant differences among sublines when analyzed with a repeated measurements ANOVA (Figure 4A). However, when EC50 values were calculated, there was a significant difference in sensitivity between WKY/NCrl vs. WKY/NHsd: 2.4±0.5⋅10^−7^ M vs. 0.9±0.4⋅10^−7^ M (P<0.05). Overall, relaxation in SHR arteries was not significantly different from WKY. However, SHR sublines showed quite variable responses, with indications for endothelial dysfunction in 2 sublines (Figure 4B). Thus, a remarkable biphasic response in mesenteric arteries from SHR/NCrl was observed. At 10^−6^ mol/L methacholine, relaxation was partially reversed in all samples of this subline. The response to methacholine was impaired in SHR/SP as compared to the other two SHR sublines (P<0.05). The EC50 values were not significantly different among SHR sublines.

### Vessel ultrastructure

**Figure 5:**
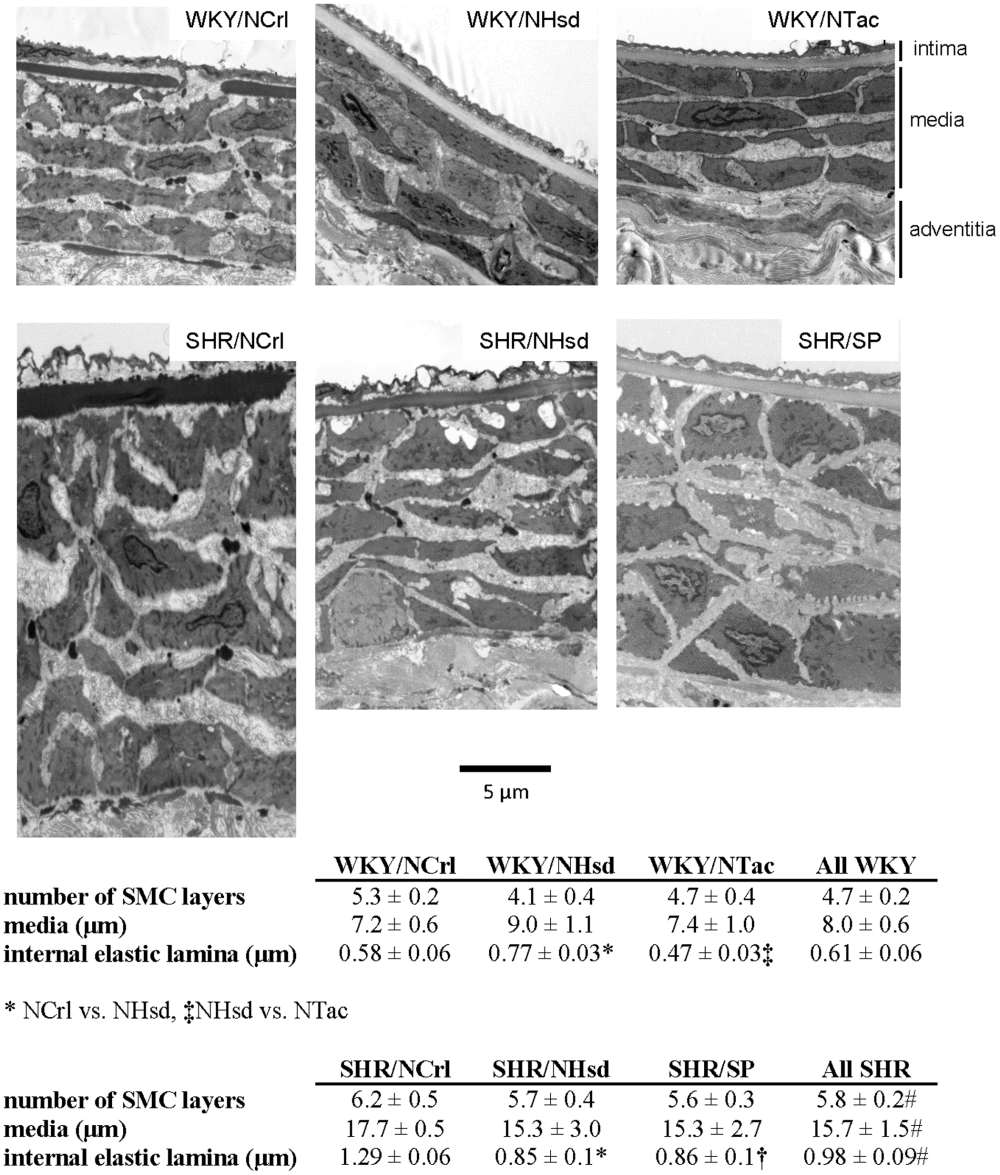
Vessel ultrastructure. Panel A depicts examples of mesenteric arteries from 5 months old rats from all groups. Panel B shows the quantification of parameters derived from EM images. From each group, 3 vessels were analyzed. SHR vessels show an increase in the number of smooth muscle cell (SMC) layers, a thicker media and a thicker internal elastic lamina.

As we observed several differences in mesenteric arterial properties, we subsequently studied vascular ultrastructure using transmission electron microscopy. In particular, we focused on the distribution and fibril diameter of collagens as these could form the basis for differences in mechanical properties. Micrographs were made from both cross sectional (not shown) and longitudinal sections of WKY and SHR mesenteric arteries at 5 months of age. Sections (Figure 5A) typically showed an intima consisting of a continuous endothelial layer, a sub-endothelial matrix, and an internal elastic lamina with occasional interruptions. The media contained several smooth muscle cell layers, embedded in extracellular matrix in which collagen fibrils and patches of elastic fibers could be identified. The media was bound by a discontinuous external elastic lamina. The adventitia mainly consisted of collagen fibrils and fibroblasts. Mesenteric arteries from the SHR/SP stood out from the other groups as we observed areas that appeared damaged, showing both intracellular and extracellular areas that were occupied by amorphous substance (not shown).

The micrographs were used to assess the number of smooth muscle layers and the thickness of the internal elastic lamina (IEL) and media. On average, SHR vessels had more smooth muscle cell layers, a thicker IEL, and a thicker media than WKY vessels (Figure 5B). There were no significant differences among WKY and SHR sublines with respect to smooth muscle cell layers and media thickness. However, the IEL thickness varied among WKY and SHR sublines.

**Figure 6:**
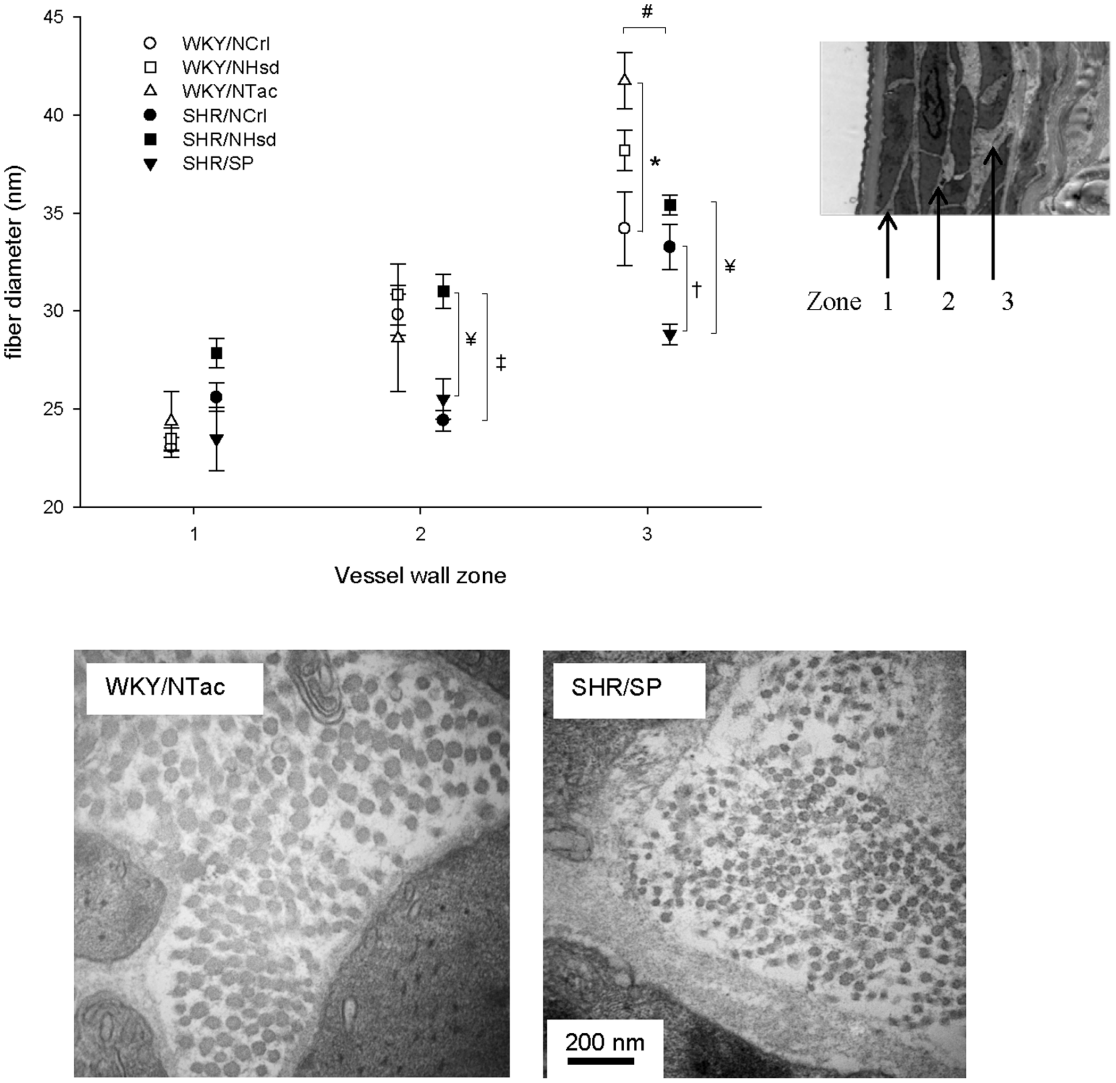
Collagen fiber size. The diameter of collagen fibers was measured in three zones of the media (panel A and insert). An increase in fiber thickness was noted from the luminal to the adventitial side of the wall. In zone 3, fibers were thinner in SHR as compared to WKY, in particular in the SHR/SP. Examples of collagen fibers in zone 3 in the WKY/NTac and the SHR/SP are shown in panel B. Data represent N = 3 in each group, at 5 months of age. On average, more than 1000 fibers were measured in each vessel. * indicates P<0.05 for WKY/NCrl vs. WKY/NTac, † SHR/NCrl vs. SHR/SP, ‡SHR/NCrl vs. SHR/NHsd, ¥ SHR/NHsd vs. SHR/SP, # WKY vs. SHR.

As differences in mechanical properties of the vessel wall could relate to differences in collagen fibril diameter, these were measured throughout the media (Figure 6A). In total, we measured approximately 25,000 collagen fibrils, divided into three zones: near the intima (zone 1), at the mid-wall (zone 2), and near the adventitia (zone 3). Overall, fibril diameter significantly increased from the luminal side towards the adventitia (P<0.05). SHR fibrils were thinner than those of WKY in the outer zone (P<0.01). Among SHR sublines, the SHR/SP stood out as its fibrils were particularly thin (see Figure 6B for comparison to WKY/NTac).

### Histology

As we observed differences in collagen fibril diameter and mechanical properties of mesenteric arteries, we speculated that this could reflect differences in the distribution of different types of collagen. We therefore stained vessel sections to determine collagen expression in the media. We found clear staining for type I collagen, mild staining for type III and type V, and clear staining for type VI collagen (Figure S3). These collagens also stained positively in the adventitia. There were, however, no obvious differences between WKY and SHR, as indicated by a comparison between the most distensible (WKY/NTac) and the least distensible vessels (SHR/SP). Decorin, a regulator of collagen fibril thickness, also did not show marked differences in staining between WKY/NTac and SHR/SP vessels.

## Discussion

To gain more insight in the pathogenesis of small artery remodeling in hypertension, we studied mesenteric arteries and aortae from WKY and SHR rats at 6 weeks and 5 months of age. We studied rats from different suppliers and included a known subline of SHR, the stroke-prone SHR (SHRSP/A3NCrl). Studying sublines of both WKY and SHR allowed us to identify common features of hypertension, but also enabled us to study the consequences of relatively small differences in the genetic background.

### Blood pressure and body weight

The heterogeneity among sublines became particularly apparent when body weight and blood pressure were measured. Large differences in blood pressure level and development over time were noted. For instance, blood pressure at 6 weeks of age may be considered a pre-hypertensive state in SHR/NHsd, but this is certainly not the case in SHR/NCrl. In the present study we relied on the tail cuff method for blood pressure measurements, which obviously has its limitations. However, blood pressure values of the WKY/NHsd and SHR/NHsd in the present study correspond very well with invasive blood pressure measurements taken from the same colony [22]. Others found also a good relation between tail cuff and invasive blood pressure measurements in WKY and SHR [23]. Yet, a drawback of the method is that a difference in pressure may exist between the tail artery and the mesenteric arteries that were studied.

Large differences in body weight were found. The high body weight of WKY/NTac, being twice that of the other WKY lines at 6 weeks of age is particularly remarkable, as is the blood pressure level in this group. The high bodyweight of the WKY/Tac, also compared to its hypertensive counterparts, has been reported by others previously [11]. With a systolic blood pressure just over 150 mmHg at 5 months of age, this animal could perhaps be considered obese and mildly hypertensive. It cannot be ruled out that housing conditions at the animal suppliers and food contribute to these differences. Thus, in the ideal case, one would breed and raise animals at one institute. However, local rules prohibited us from doing so. It is also possible that this particular subline matures at a faster rate than other WKY sublines. The potential problem of differences in body weight and maturation could be that comparisons of WKY to SHR arteries are obscured by such differences, as we found that mesenteric arteries enlarge with maturation, and also become more distensible with maturation in WKY. In this respect it is noteworthy that in another model of hypertension both cardiac and vascular remodeling are time-dependent [24], [25]. Thus, differences in the dynamics of remodeling may be present among sublines of rats.

### Inward remodeling: yes and no

The concept that resistance vessels show inward remodeling in hypertension is based on the observation that lumen diameters are reduced while the amount of wall material is not necessarily altered in comparison to normotensive counterparts [26]. In the present study, mesenteric artery lumen diameters did show a tendency to be smaller in SHR, but this did not reach statistical significance. Yet, comparison of diameters between individual sublines of WKY and SHR could suggest a reduction in diameter with hypertension. The 15% smaller diameter in SHR/NHsd as compared to WKY/NTac could be interpreted as inward remodeling. Yet, vessels from SHR/NCrl are similar in size to WKY/NCrl (+1%), which is in good agreement with published data [9]. Therefore, conclusions on remodeling depend on the particular sublines of WKY and SHR that are compared.

We found that mesenteric arteries in all groups enlarge with maturation. In vessels from WKY but interestingly not SHR, this is accompanied by an increase in distensibility. In any case, in the time frame of the current study, the diameter of neither SHR nor WKY vessels decreased with the rise in blood pressure that develops over time, as the term inward remodeling would suggest. Rather, there is a tendency for a smaller diameter in vessels from SHR that is present already at 6 weeks of age. This is accompanied by a hampered increase in distensibility in SHR, which could perhaps contribute to a smaller diameter. We therefore propose that during development, inward remodeling should be seen as a reduced outward remodeling, rather than true inward remodeling. Future studies into the development of the vessel structure, including vessels from very young animals and at old age, could further elucidate this.

### Endothelial dysfunction

As endothelial dysfunction is commonly associated with hypertension, we tested responses to methacholine in mesenteric arteries. This test focusses on endothelium-dependent relaxation only, and does not reflect other possible differences such as inflammatory activation or coagulable state. It has been noted that endothelial function may be impaired, unaltered or even enhanced in SHR as compared to WKY, depending on vessel type, age, and experimental conditions [16]. In the present study we found that, similar to comparisons of blood pressure and vascular diameter, conclusions regarding endothelium-dependent relaxation between WKY and SHR also depend on the chosen sublines. A clearly impaired endothelium-dependent relaxation was found in mesenteric arteries from SHR/SP, but not in SHR/NHsd. The SHR/NCrl showed a partial reversal of contraction at an intermediate dose of methacholine. The underlying mechanisms for these differences are not immediately clear from these experiments, as endothelial function is regulated by many factors. Methacholine can induce dilation via nitric oxide, endothelium-derived hyperpolarizing factors, prostaglandins, and even release endothelium-derived contracting factors. Nitric oxide may be scavenged by reactive oxygen species, particularly in SHR vessels. Additional experiments would be needed to sort this out. Taken together, comparison of endothelial responses in mesenteric arteries from sublines of WKY with sublines of SHR can either substantiate or refute the notion of endothelial dysfunction in SHR.

### Vascular stiffness relates to ultrastructure

We further explored the basis of vascular remodeling in SHR by calculating stress-strain relationships and comparison of vessel ultrastructure. The stress-strain relationships indicated an increased stiffness of the elastic component in mesenteric arteries of six weeks old SHR. Although one should be careful to attribute vessel mechanics to specific matrix molecules, these data support the suggestions of Briones et al. [8] and Arribas et al. [27] regarding the role of elastin in the development of hypertension. They found excessive and aberrant elastin deposited in SHR vessels during perinatal development. Differences between WKY and SHR vessels could be eliminated by elastase treatment. Their work indicates that abnormalities in elastin deposition at a very young age limit vessel expansion and possibly contribute to hypertension in later life. In this respect, it has been shown that elastin, but also other matrix components such as collagen I are deposited in early life and have a very low turnover rate under normal conditions [28]. We also found that mesenteric arteries of SHR exhibit an increase in the thickness of the internal elastic lamina at 5 months of age, as observed with electron microscopy. At this point in time, the stiffness attributed to elastin was no longer significantly increased. These findings are not necessarily discordant, since the parameters that we used to calculate elasticity reflect intrinsic material properties, not amount.

Medial collagen fibrils were found to increase in diameter from the lumen towards the adventitia. Such a phenomenon has been noted previously in human aorta [29]. The basis and the implications of this are currently unknown. The stress-strain relationships suggested that collagen stiffness was not consistently altered in mesenteric arteries from SHR. However, large differences were found when comparing WKY and SHR sublines. Comparison of the extremes, the WKY/NTac vs. the SHR/SP, showed that the more distensible vessels of the WKY/NTac show a late collagen hook-on and high collagen stiffness. In contrast, vessels from the SHR/SP show an early collagen hook-on and a relatively low collagen stiffness. In the analysis of collagen fibril thickness, the WKY/NTac showed the thickest fibrils, whereas the SHR/SP had the thinnest fibrils. Thus, these data are consistent with the notion that thinner collagen fibrils are less stiff, and fibril thickness represents a major determinant in stress-strain relationships of collagen fibils [30].

The presence of thinner collagen fibrils could relate to differential expression of collagen types. However, we found no obvious differences in the distribution of collagen types I, III, V and VI in the media of mesenteric arteries from WKY/NTac vs. SHR/SP. We also stained for decorin, a known regulator of collagen fibril formation [31]. However, also this did not reveal a clear explanation for the reduced fibril thickness. An alternative explanation for the reduced fibril thickness in SHR/SP could be that there is an increased turnover of collagen, as thinner fibrils are indicative of repair processes [32].

### Aortae

Although we did not study the aortae as extensively as the mesenteric arteries, several of the findings on these vessels were consistent with those made in the small mesenteric arteries. These include the increase in wall-to-lumen ratio in aortae of SHR as compared to WKY, and the lack of consistent differences in vessel size and stiffness between SHR and WKY. The fact that aortae from the SHR/SP showed the largest wall CSA and wall-to-lumen ratio seems to be consistent with the observation that this group showed the highest blood pressure.

### Conclusions

This study shows that sublines of WKY and SHR display differences in a number of parameters. These include blood pressure, mesenteric small artery dimensions and mechanical properties, endothelial function, vessel wall ultrastructure, but also body weight. The result of these differences among sublines is that comparisons between WKY and SHR can lead to completely opposite conclusions with respect to the pathophysiology of vascular function and structure in hypertension. Thus, while an increase in wall-to-lumen ratio and an increase in wall thickness are typical features of all SHR sublines studied herein, inward remodeling, increased stiffness, or impaired endothelium-dependent relaxation are subline-dependent. These findings may help to explain the discrepancy in literature on these topics. The results also suggest that relatively small differences in genetic background, and possibly differences in housing, are associated with profound differences in vascular pathophysiology in hypertension. We believe that this does not disqualify the SHR as a model of hypertension, but caution is warranted when data from a particular subline of WKY and SHR are extrapolated to hypertension in general. We therefore recommend to include more than one subline of WKY and SHR, or include different models of hypertension when studying the pathophysiology of small artery remodeling.

## Supporting Information

Figure S1 **Vascular distensibility in WKY and SHR sublines.**
(DOC)

Figure S2 **Aortic mechanical properties and dimensions.**
(DOC)

Figure S3 (DOC)
