# Supplementary material for: Heterogeneity in Arterial Remodeling among Sublines of Spontaneously Hypertensive Rats
Source: PLoS One. 2014 Sep 24;9(9):e107998. doi: 10.1371/journal.pone.0107998 (PMC4175999; doi:10.1371/journal.pone.0107998)
Supplement: Figure S1 — Vascular distensibility in WKY and SHR sublines. (DOC) [file pone.0107998.s001.doc]

**Figure S1.**

**Vascular distensibility in WKY and SHR sublines.** Diameters were measured on cannulated mesenteric arteries, and normalized to the lowest pressure level to eliminate differences in initial diameter. Vessels were measured under fully dilated conditions. Each group consists of n≥5 animals, where 2 vessels from each animal were measured and averaged. * NCrl vs. NHsd, † NCrl vs. SP
