# Supplementary material for: Heterogeneity in Arterial Remodeling among Sublines of Spontaneously Hypertensive Rats
Source: PLoS One. 2014 Sep 24;9(9):e107998. doi: 10.1371/journal.pone.0107998 (PMC4175999; doi:10.1371/journal.pone.0107998)
Supplement: Figure S2 — Aortic mechanical properties and dimensions. (DOC) [file pone.0107998.s002.doc]

**Figure S2.**

**A**

B

|  | WKY/NCrl | WKY/NHsd | WKY/Tac | SHR/NCrl | SHR/NHsd | SHR/SP |
| --- | --- | --- | --- | --- | --- | --- |
| Diameter (mm) | 2.3±0.1 | 2.3±0.1 | 2.4±0.1†‡ | 2.5±0.2 | 2.2±0.05* | 2.3±0.02 |
| Wall CSA (mm^2^) | 0.75±0.17 | 0.70±0.12 | 1.00±0.18†‡ | 0.83±0.03 | 0.85±0.05 | 1.14±0.12†¥ |
| Wall/lumen | 0.32±0.04 | 0.32±0.03 | 0.31±0.02 | 0.33±0.01 | 0.34±0.01 | 0.36±0.02¥ |

* NCrl vs. NHsd, † NCrl vs. NTac, ‡NHsd vs. NTac, † NCrl vs. SP, ¥ NHsd vs. SP

**Aortic mechanical properties and dimensions.** A) Segments of the thoracic aorta were mounted in a wire myograph and stretched in a stepwise manner. Distension and force were recorded. Stress-strain values were calculated based on distension, force, and wall thickness. There was no significant difference between WKY and SHR, nor among individual sublines. B) Calculated diameters are shown for an equivalent pressure of 100 mmHg. The wall cross sectional area was determined in sections, fixated without pressure. The wall to lumen ratio is given for aorta’s at slack length. Only the wall-to-lumen ratio was systematically larger in SHR as compared to WKY.
