# Supplementary material for: Heterogeneity in Arterial Remodeling among Sublines of Spontaneously Hypertensive Rats
Source: PLoS One. 2014 Sep 24;9(9):e107998. doi: 10.1371/journal.pone.0107998 (PMC4175999; doi:10.1371/journal.pone.0107998)
Supplement: Figure S3 — (DOC) [file pone.0107998.s003.doc]

**Figure S3.**


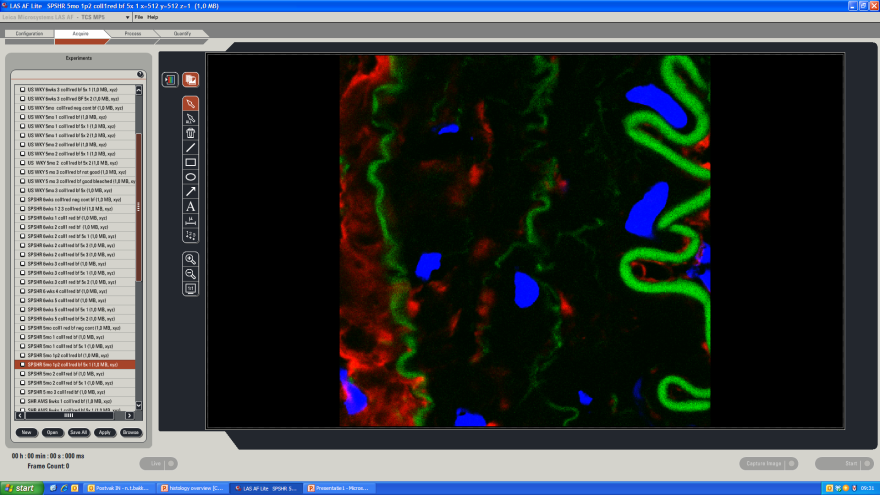

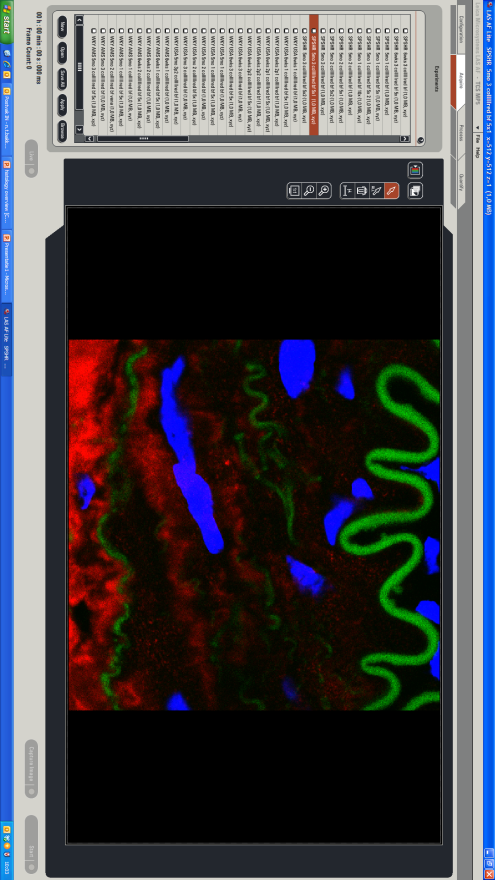

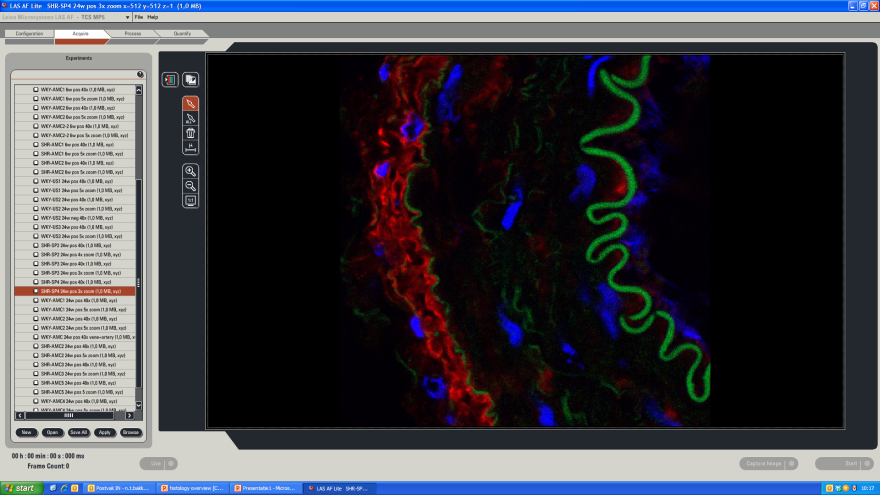


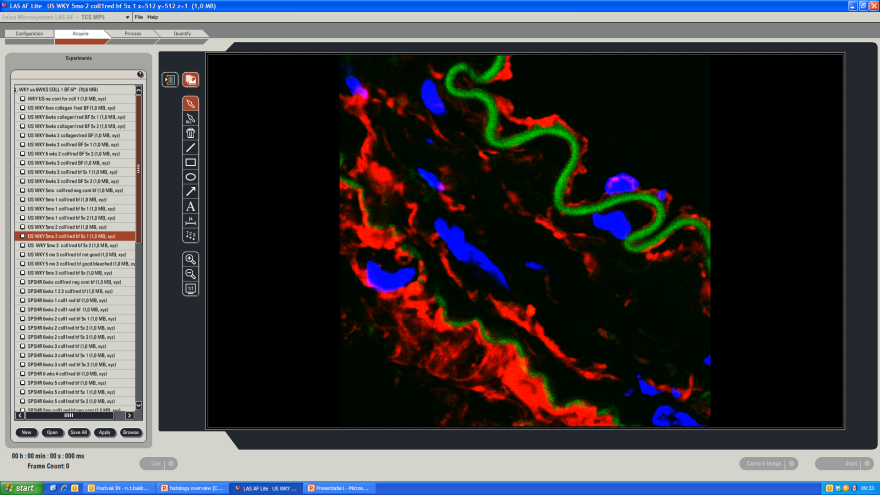

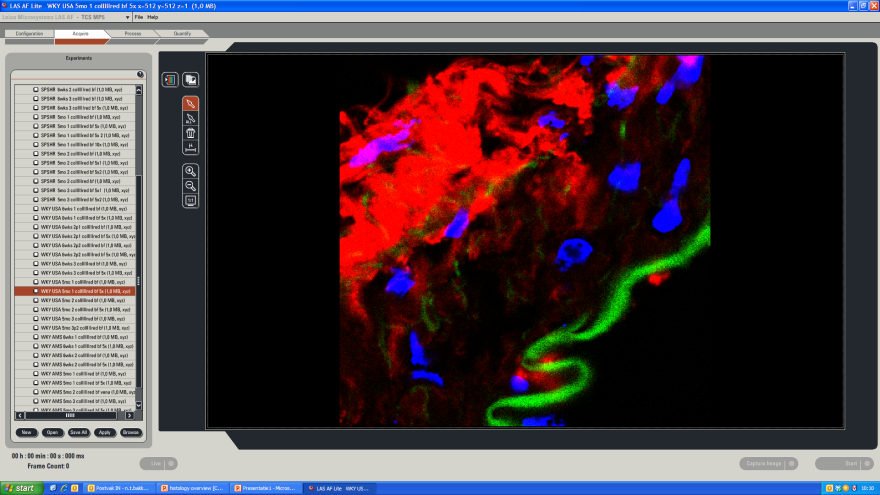

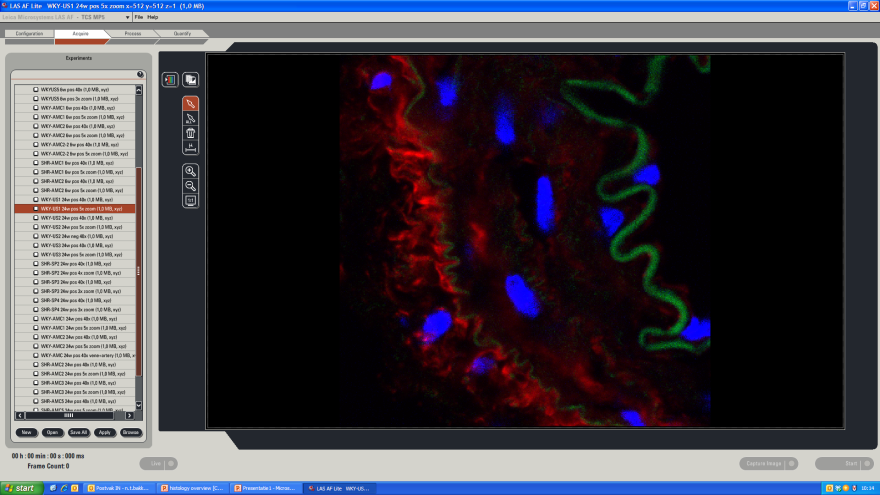

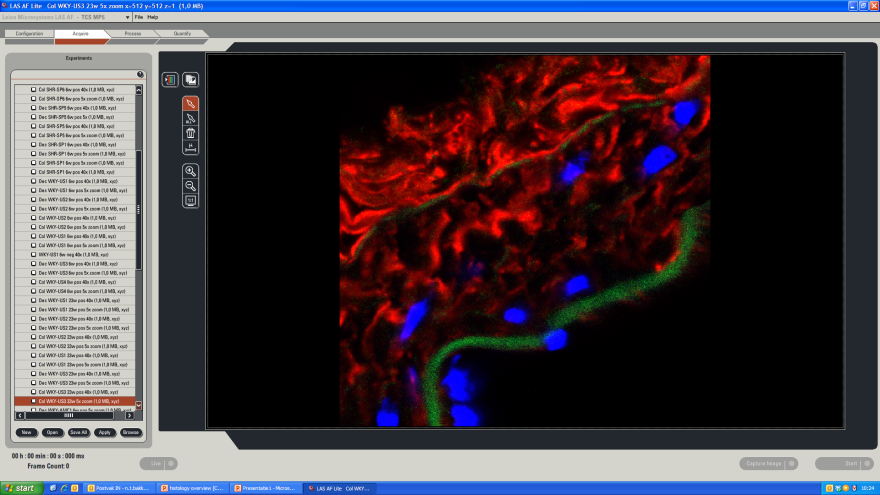

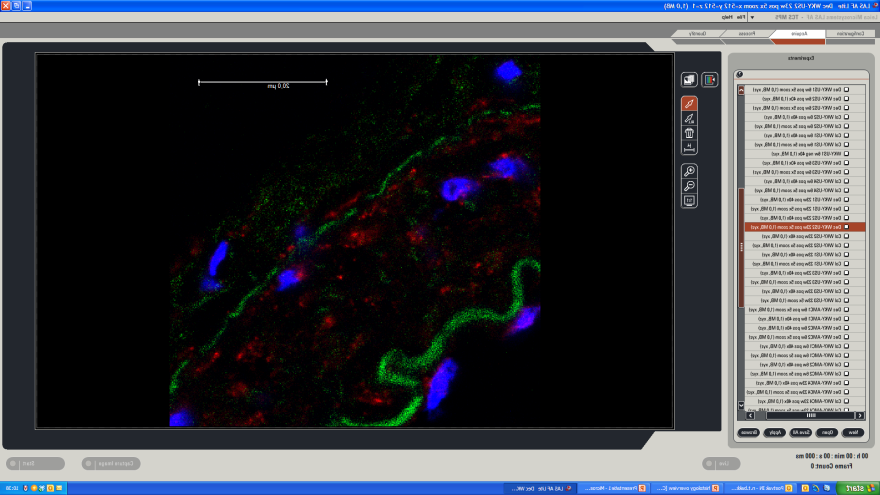


20 μm

WKY/NTac

Col I

Col III

Col V

SHR/SP


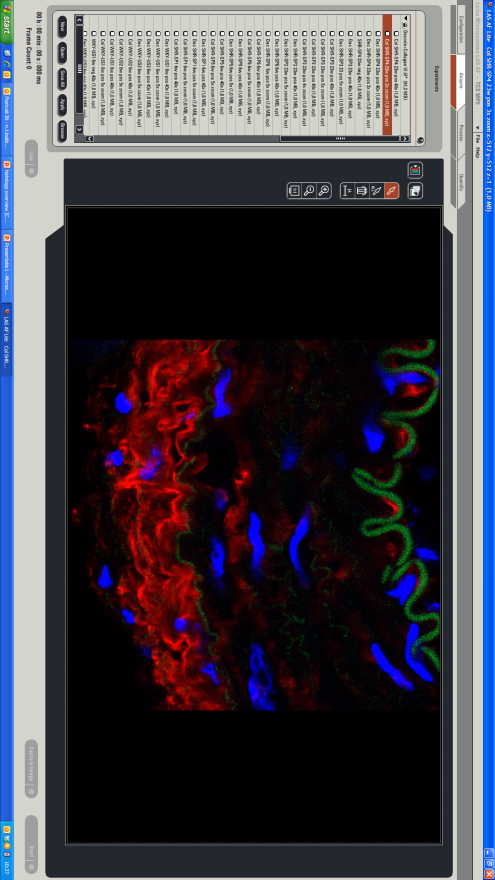

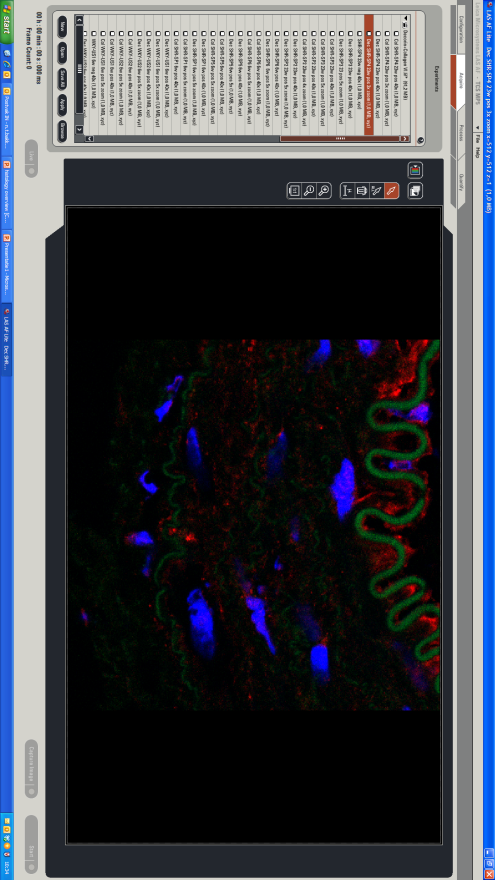


Col VI

Decorin

Immunostaining for collagens type I, III, V, VI, and decorin (red) in arteries from the most distensible (WKY/NTac) and least distensible group (SHR/SP) at 5 months of age. No clear differences in distribution pattern were noted. Nuclei were stained with bisbenzimide (blue). Autofluorescence of elastin is depicted in green.
